# Highthroughtput analysis of behavior for drug discovery

**DOI:** 10.1016/j.ejphar.2014.11.047

**Published:** 2015-01-12

**Authors:** Vadim Alexandrov, Dani Brunner, Taleen Hanania, Emer Leahy

**Affiliations:** PsychoGenics Inc., Tarrytown, New York, USA

## Abstract

Drug testing with traditional behavioral assays constitutes a major bottleneck in the development of novel therapies. PsychoGenics developed three comprehensive highthroughtput systems, SmartCube^®^, NeuroCube^®^ and PhenoCube^®^ systems, to increase the efficiency of the drug screening and phenotyping in rodents. These three systems capture different domains of behavior, namely, cognitive, motor, circadian, social, anxiety-like, gait and others, using custom-built computer vision software and machine learning algorithms for analysis. This review exemplifies the use of the three systems and explains how they can advance drug screening with their applications to phenotyping of disease models, drug screening, selection of lead candidates, behavior-driven lead optimization, and drug repurposing.

## 1. Introduction

Neuropsychiatric, developmental and neurodegenerative disorders are complex and involve multiple neuronal circuits. Target-based approaches have, for the most part, failed to deliver meaningful treatments, whereas phenotypic screening has proved more successful. In the period between 1999 and 2008, 75 first-in-class drugs with novel mechanism of action were approved. Of the first-in-class drugs, 28 were discovered using phenotypic screening vs. 17 using target-based approaches. Specifically in CNS, 7 of the 8 first-in-class drugs approved were discovered using phenotypic screening (Swinney and Anthony, 2011).

It is not surprising, therefore that many of the most efficacious drugs, especially in psychiatry, have multiple targets and were discovered by serendipity (observing how an animal's behavior was altered in response to the drug). Since the goal of any neuropsychiatric drug is to impact behavior, PsychoGenics has industrialized “serendipity” with its behavior-based technologies.

**Figure 1:**
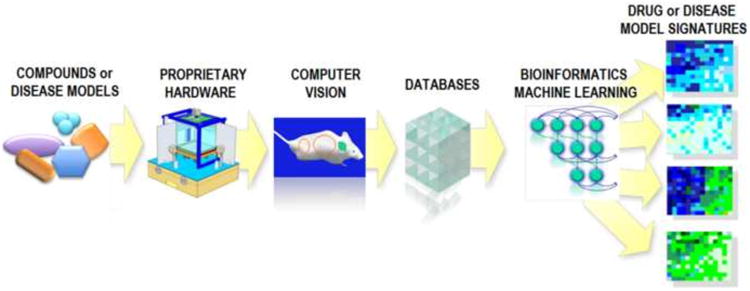
SmartCube^®^ combines behavioral neurobiology insight integrated with advances in robotics and computer vision (video capture and analysis) and the power of bioinformatics to process and analyze massive temporal and vectorial datasets using probability causal inference algorithms. SmartCube^®^ is a platform that provides a sequence of challenges to a mouse, extracts more than 2000 features during a session and using proprietary bioinformatics detects the potential of compounds to treat psychiatric disorders in an unbiased way by comparing their complex behavioral profiles with those from a proprietary reference database.

PsychoGenics' proprietary behavior-based technologies, also known as the *SmartCube*^®^*, NeuroCube*^®^ and *PhenoCube*^®^ systems, combine behavioral neurobiology insight integrated with advances in robotics and computer vision (video capture and analysis) and the power of bioinformatics to process and analyze massive temporal and vectorial datasets using probabilistic causal inference algorithms (Fig. 1). The technologies offer numerous distinct advantages over current behavioral assessment including the following:

**High throughput** - can screen tens of thousands of compounds for CNS activity and identify those with a behavioral profile that reverses a disease model phenotype or is reminiscent of drugs that treat a specific neuropsychiatric disorder;
**High content** – thousands of features are collected and proprietary bioinformatics algorithms are employed to detect subtle phenotypic differences associated with a disease model or drug effect.
**Unbiased** – Computer vision algorithms and bioinformatics eliminate human intervention and subjectivity.

PsychoGenics uses its platforms at all stages of drug discovery as described below, to identify novel treatments addressing major unmet neuropsychiatric disorders that are unlikely to be found by other means. Platform applications include:

- Screening representative compounds from diverse CNS libraries. This approach is agnostic to compound mechanism of action;
- Re-purposing compounds that are discontinued (for reasons other than safety) or currently being developed for other non-CNS indications;
- Screening target-focused compounds to determine the therapeutic utility of a target or identifying a preferred chemotype;
- Assessing compound combinations (i.e. determining the efficacy of a combination of novel compounds or a novel compound combined with an existing marketed drug);
- Lead optimization.

Using this approach, PsychoGenics has identified several drug candidates at various stages of clinical and preclinical development on its own and in partnership with other companies.

## 2. The SmartCube^®^ System

The SmartCube^®^ system is a high-throughput automated behavioral platform that presents a sequence of challenges to a mouse through its customized hardware, extracts more than 2000 features per session, and, using proprietary bioinformatics, and detects the potential therapeutic efficacy of compounds.

SmartCube^®^ employs computer vision and mechanical actuators to detect spontaneous and evoked behavior eliciting responses through anxiogenic and startling stimuli. Behavioral readouts include locomotion, trajectory complexity, body posture and shape, simple behaviors and behavioral sequences (Brunner et al., 2002; Houghten et al., 2008; Roberds et al., 2011). Supervised machine learning algorithms are used to analyze the collected features. Although approximately ½ million datapoints are collected per mouse per session, behavioral definitions, machine learning techniques and smart voting under uncertainty, are used to reduce this dataset to ∼ 2000 target features.

**Figure 2:**
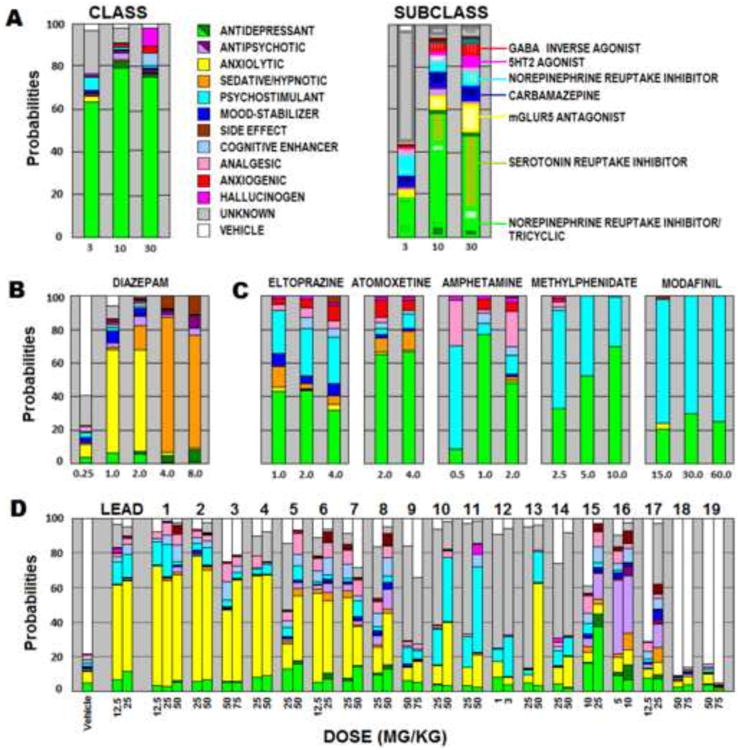
Drug signatures and lead optimization in SmartCube^®^. **A:** The signature of an mGlurR2/3 antagonist in SmartCube^®^ showing a strong antidepressant signal at the class level (the green bar height represents the strength of the antidepressant signal), and a transition from tricyclic to SSRI at the Subclass level. The legend show all classes some subclasses present in PGI proprietary database. **B:** Dose-response signature of diazepam in SmartCube^®^ ranging from inactive, to anxiolytic and finally to sedative hypnotic as the dose increases. **C:** Eltoprazine exhibits a mixed signature (green-antidepressant and cyan- psychostimulant) that is similar to other stimulant and non-stimulant drugs currently used in the treatment of ADHD. **D:** A lead compound was found combing through available chemical libraries. A desired signature of anxiolytic (yellow) and psychostimulant signals (cyan) is then sought after by generation of new analogs. Some compounds show a very similar signature (1 and 2) and others show activity but signatures that deviate from the lead. Compounds 15-17, for example show an antipsychotic signal (purple) and some others are completely inactive (18-19), despite being chemically similar to the lead.

PsychoGenics' proprietary supervised machine learning methodology, derived from minimization of Bayesian misclassification probability, similar in spirit to Support Vector Machines, is used to train a classification algorithm that reliably maps behavioral features for each drug to its corresponding biological response “label” (e.g. CNS Indication or Mechanism of Action). The original feature space undergoes non-linear transformation using a proprietary semi-blind source separation variant of Independent Component Analysis to minimize “overcounting”, during calculation of the contribution of overrepresented original features, and reduce the effective (new) feature dimensionality. The output of the resulting classification algorithm is a probability distribution over the chosen set of labels which, in addition to a specific biological response, predicts quantities such as “unknown activity” (difference from vehicle not attributable to any specific feature patterns in the training set) as well as “total activity” of the drug (Fig. 2).

Two major types of analyses are routinely conducted: Class and Subclass. For Class and Subclass analyses, a reference data set has been built from hundreds of drug doses grouped in multiple drug classes plus a vehicle class. Dose responses for the reference drugs were constructed using multiple doses targeting both efficacious doses as well as doses that exhibit side effect profiles in mice. The Class analysis uses labels and corresponding drugs that are currently in the market or have been clinically validated for a specific therapeutic indication. The Subclass analysis uses labels and a larger set of compounds selected from both marketed drugs and compounds validated for specific therapeutic uses. The reference databases are continually expanding with the addition of novel therapeutics and new proprietary databases are currently in development.

Novel compounds can be tested in SmartCube^®^ system and the results can then be compared to the signatures of reference compounds in PsychoGenics' database. Multiple analyses of the data are performed to quantitatively produce independent predictions of drug class, and drug subclass. The system, therefore, can, in an unbiased way classify compounds according to the therapeutic potential by comparing their complex behavioral profiles with those from a proprietary reference database.

The results for the class and subclass analyses are presented as standardized bar charts with percentages that sum to 100 for each dose. The results of the classification at the drug level are presented as individual similarities. An example of the output of a typical classification is shown in Fig. 2A.

Fig. 2B shows a different use of the system, as a full profile appears when a large dose response is run, in this case for diazepam. The drug goes from inactive at 0.25 mg/kg to anxiolytic between 1.0 to 2.0 and sedative at higher doses. In this way, therefore, a therapeutic window and complete profile can be established for any drug. An interesting characteristic of the total pharmacological activity represented by the height of the colored bar is that it captures all beneficial, neutral, detrimental and unknown effects of the drug, so it continues to grow as the dose increases, but the color profile changes indicating the changing nature of the pharmacological action.

Fig. 2C depicts one of the first projects that benefited from the SmartCube^®^ system. Different psychostimulant drugs are shown and compared against Eltoprazine. Despite Eltoprazine being from a very different class (a partial 5HT1A/1B agonist) it showed similarity to drugs used in Attention Deficit Hyperactivity Disorder (ADHD). Using these results and other preclinical experiments that confirmed activity of this compound in attenuating hyperactivity and impulsivity in various animal models, PsychoGenics conducted a proof-of-concept study in adults with ADHD. The study showed both doses tested (5 mg bid and 10 mg, bid) significantly improved ADHD symptoms using the ADHD rating scale (The Foundation for Medical Practice Education, www.fmpe.org, 2008) as compared to placebo (p < 0.003 and 0.037, respectively).

### 2.1 Lead optimization

Drug development involves a time consuming lead optimization process that depends on laborious and time consuming structure activity relationship models. Using an *in vivo* readout allows fast assessment of alternative modifications to a pharmacophore (Houghten et al., 2008). Fig. 2D shows an example from one of PsychoGenics' internal drug development programs in which 1,400 compounds were selected from commercially available libraries. A lead was found based on its interesting signature in SmartCube^®^ and confirmation of therapeutic effects in standard tests. As the lead compound had a short half-life a number of analogs were synthesized and ran through SmartCube. The quick feedback allowed chemists to quickly proceed through the structure activity relationship modeling and focus on changes to the pharmacophore that preserved the desired phenotypic signatures (Brunner et al., 2012).

Whereas in this project, the mechanism of action was unknown for much of the development (a phenotypic approach), other similar projects use target-specific libraries of known mechanism of action, even combination of compounds of different mechanism of action in search of specific synergies.

### 2.2 Quantitative Assessment of a Disease Phenotype and its Progression

The more than 2000 behavioral features collected from SmartCube^®^ can also be analyzed using machine learning algorithms to determine the feature set that best represent a disease model and differentiate it from control.

#### Feature analysis: de-correlation and ranking

Many of the features from SmartCube^®^ are correlated (e.g. rearing counts and supported rearing counts). Therefore, PsychoGenics forms statistically independent combinations of the original features (further referred to as *de-correlated features*) that discriminate between the two groups more effectively. Each de-correlated feature extracts information from the whole cluster of the original features, so the new feature space has lower dimensionality.

Next, PsychoGenics applies a proprietary feature ranking algorithm to score each feature's discrimination power (ability to separate the two groups, e.g. control and disease). Ranking is an important part of the analyses because it weighs each feature change by its relevance: if there is a significant change in some irrelevant feature measured for a particular phenotype, the low rank of this feature will automatically reduce the effect of such change in the analyses, so we don't have to resort to the conventional “feature selection” approach and discard information buried in the less informative features. The ranking algorithm can be applied to either the original or the new features to gain insight about the key control-disease differences.

#### Feature analysis: quantitative assessment of Disease Phenotype

**Figure 3:**
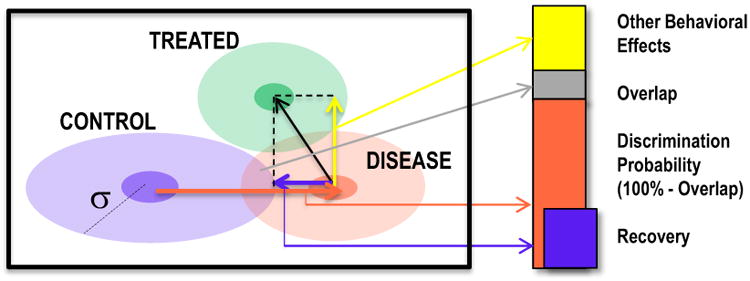
Visualization of a binary discrimination in the ranked de-correlated feature space. **Left**. The two highest ranked de-correlated features form the 2D coordinates plane for visualization purposes. Mice from the control group are shown as a blue “cloud” and mice from the disease group are plotted in red. From the overlap between the two clouds we can derive discrimination probability = 1 - overlap, which measures how reliably a classifier can be trained to discriminate between the two groups with zero corresponding to 100% overlap (and no ability to distinguish the two groups above the chance level) and 100% meaning error free discrimination. **Right**: The “recovery signature” graph summarizing the recovery analysis. The overlap (gray) and discrimination probability (red) sum up to 100%. Recovery (blue) ranges from zero up to the discrimination probability value. “Other behavioral effects” (yellow) represent drug effects in an orthogonal direction.

In the new feature space, the overlap between the “clouds” (Gaussian distributions approximating the groups of mice in the ranked de-correlated features space) serves as a quantitative measure of *separability (“distinguishability”)* between the two groups (Fig. 3). For visualization purposes, we plot each cloud with its semi-axes equal to the one standard deviation along the corresponding dimensions.

A third group, “treated”, can be plotted in the same coordinate system that best discriminates Control and Disease, as shown in Fig. 3. The drug treatment effect can then be represented as a combination of two components: one along the direction of the “recovery line” (the line connecting the centers of the Control and Disease clouds) shown as a blue arrow, and the component orthogonal to (“pointing away” from) that direction shown as a yellow arrow. The relative length of the “recovery” (blue) arrow with respect to the Control-Disease distance can then be interpreted as the “recovery due to the drug”, whereas the relative length of the “other effect” (yellow) arrow represents feature changes that move the Treated group away from the Control group. The summary of this analysis can be effectively represented as a bar graph (right pane in Fig. 3) which we typically refer to as the recovery signature.

**Figure 4:**
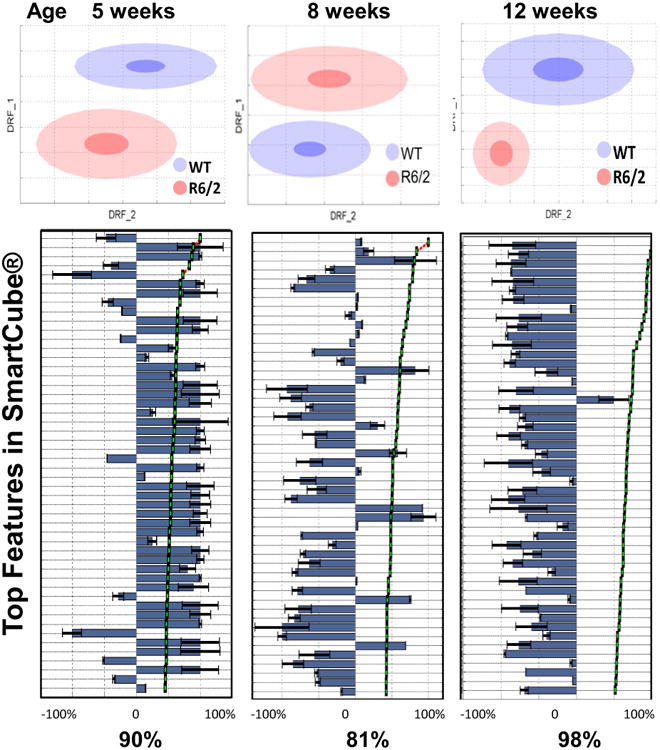
Disease signature of the R6/2 mouse model in SmartCube at three different ages showing age-specific signatures. Features that were increased in the 5 week old mice are plotted towards the right of the scale (right of the zero value on the x-axis) including mainly measures of increased activity. In the older mice, features are decreased (towards the left of the zero value on the x-axis), showing the beginning of the hypoactive terminal phase.

Fig. 4 shows an example of the ranked features that separate R6/2 mice, a model of Huntington's disease, from its wild type control and the binary discrimination in a 2D cloud. We ordered the features according to the rank obtained at each age, and could see very strong discrimination against the wild type control. However, the features that were different at 5 weeks of age where different than those affected at 8 and 12 weeks when pathology starts to be apparent. Indeed, whereas the signature shows an increasing hypoactive phenotype at the older ages, it comprised a hyperactive phenotype at the 5 week mark, possibly signifying a prodromal phase of the disease.

## 3. The NeuroCube^®^ System

The NeuroCube^®^ system is a fully automated *in-vivo* high-throughput platform that is used to assess motor performance and gait in mice and rats. The system utilizes computer vision and machine learning algorithms to automatically track every locomotion detail of an animal and measure parameters of gait geometry, gait dynamics as well as non-locomotion behaviors.

Subjects are allowed to freely walk for 5 minutes in the NeuroCube^®^ system. Digital videos of the subjects are captured and processed through computer segmentation algorithms. The resulting fitted parameters are then analyzed to extract clips of locomotor behavior. Those clips are further analyzed to extract information about gait geometry (stride length, step length and base width) and gait dynamics (stride duration, step duration and swing duration). In addition, the system provides data relating to the following:

- Average Speed of the animal
- Paw Image intensity, paw contact area, perimeter of contact zone, and paw diameter
- Paw Position relative to the center of the body is registered.
- Body Position as it pertains to movement of the subject
- Rhythmicity and limb coordination

The sensitivity of the NeuroCube^®^ system to capture subtle gait changes allows it to objectively quantify disease progression in various rodent models of neurodegenerative and neurodevelopmental disorders as well as in preclinical models of pain and injury.

Classification algorithms are used to define and rank the most dominant features that define the disease phenotype. Complex bioinformatics are employed to calculate the discrimination probability between the control and disease animals which would help determine onset of the disease phenotype for pharmacological interventions.

Chronic neuropathic pain remains a widespread disorder within the health sciences. Lack of translation between preclinical and clinical research continues to be a challenging problem in this area as in other neuroscience domains (Brunner et al., 2011; Munafo et al., 2014; Taneja et al., 2012). Most of the preclinical models of neuropathic pain that use evoked thermal or mechanical single endpoints provide poor predictive validity as the majority of human clinical pain is considered spontaneous in nature. By using the NeuroCube^®^ system, many behaviors of a freely moving animal are captured without the need of thermal or mechanical manipulations. Using sciatic or spinal nerve ligation models the system can define the behaviors that best define symptoms of neuropathic pain by ranking the features that show discrimination between a sham and a ligated animal for example. The algorithms can then be applied to assess the efficacy of therapeutic compounds to reverse these pain features. It is possible that using this automated, less subjective approach can provide a better translational tool for neuropathic pain research.

**Figure 5:**
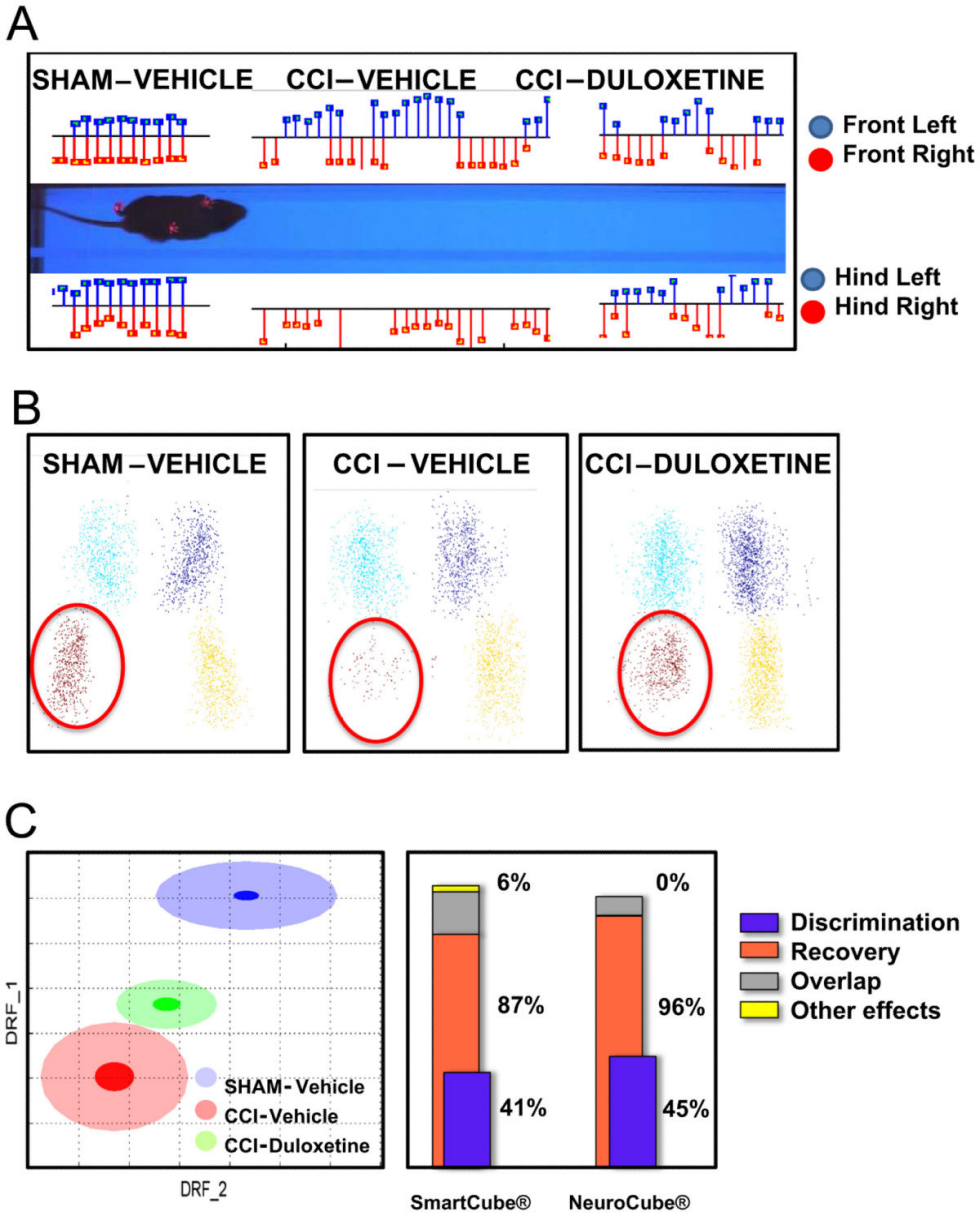
**A.** Paw image intensity in sham, chronic constrictive nerve injury (CCI) and duloxetine-treated CCI mice. **B.** Pooled paw position of sham, CCI and duloxetine-treated CCI mouse. The CCI mouse shows avoidance to place the injured paw on the platform of the NeuroCube^®^ system. Treatment with duloxetine restores this behavior. **C.** Cloud graph visualization of the sham, CCI and CCI + duloxetine groups relationships in NeuroCube (left) and the recovery signature graphs in both NeuroCube and SmartCube (right). The CCI-duloxetine cloud (green) is between the sham and CCI groups suggesting recovery. The quantification of this effect shows 41% recovery. Recovery as measured by SmartCube is similar, at 45%.

Fig. 5 shows the effects of acute administration of duloxetine on paw placement in the chronic constrictive nerve injury model of neuropathic pain (Bennett and Xie, 1988; Sommer et al. 1997*)*. Whereas a sham-injured mouse walks on all four paws in a rhythmic and symmetric way, a nerve injured mouse shows clear avoidance to place the paw of the ligated side (left hind paw) on the platform (Fig. 5A & B). Furthermore compensation in gait is seen as the mouse adds more pressure on the front paws. Following administration of duloxetine, a partial recovery of gait is seen and the mouse is able to place the injured paw on the platform while locomoting. These effects are in line with the mechanical allodynia test showing that duloxetine increases paw withdrawal threshold in this model (Joshi et al., 2006; Le Cudennec and Castagne, 2014).

Fig. 5C shows quantitative analyses for the effects of duloxetine on recovering of pain features in both SmartCube^®^ and NeuroCube^®^ systems in the mouse chronic constrictive nerve injury model and in a standard mechanical allodynia model. The model mice and sham mice could be differentiated with 87% accuracy in SmartCube^®^ and 96% accuracy in NeuroCube^®^. Duloxetine reduced such separation by 41% and 45% in SmartCube^®^ and NeuroCube^®^, respectively.

## 4. The PhenoCube^®^ System

The PhenoCube^®^ System provides an environment where disease models or treatments can be assessed over several days. Groups of mice are challenged in ways that allow the system to detect social, circadian, motor, and cognitive behaviors, hallmarks of most neuropsychiatric disorders. The system acquires a broad range of different measures that span multiple disease-relevant domains (i.e., cognition, locomotor activity and circadian patterns), and thus can efficiently capture the complexity of the behavioral phenotype. Complex computer vision and automation eliminate any subjectivity and together with proprietary data mining algorithms can detect subtle changes even early in the progression of a disease phenotype. The cognitive challenges presented in the environment can be employed to identify compounds with potential to treat cognitive impairment associated with disorders such as schizophrenia, Alzheimer's disease, and ADHD.

**Figure 6:**
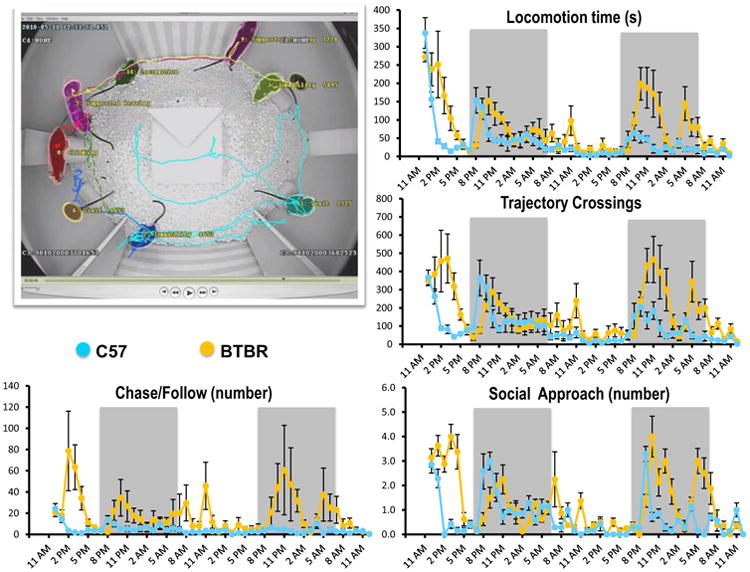
PhenoCube^®^ system. An inside view of the system showing visual cues and objects for assessment of motor behavior. The circular openings are the entry points and associated antennae corresponding to the Intellicage^®^ system. Two wild type strains that show very different behavioral patterns in many tests were used to assess the ability of PhenoCube^®^ to measure dyadic social behavior. BTBR mice showed reduced activity throughout days and nights (Mann Whitney, p < .002). A simple measure of casual encounters, crossing of locomotor trajectories, showed a very similar pattern (Mann Whitney, p < .002). True active social approaches (approach follow by an interaction), however, had a different pattern with only a slight non-significant trend toward lower approaches in the BTBR mice (Mann Whitney, p > .18). Other behaviors, likely of a more aggressive nature, following and chasing, showed again a higher frequency in the C57 mice than in the BTBR (Mann Whitney, p < .02). These results are consistent with the reputation of the C57 as an aggressive strain but somehow inconsistent with lower sociality in the BTBR mice.

PsychoGenics developed the PhenoCube^®^ through hardware modifications of Intellicage units (New Behavior, AG, Zurich, CH) and addition of custom-built computer vision hardware and software. This system enables behavioral phenotyping of group-housed mice within a home-cage-like environment over multiple days with minimal experimenter interruption, thereby allowing comprehensive capture of the natural behavioral rhythms of the subjects. A day/night Camera mounted on top of the cage allows recording of the subjects and the use of PsychoGenics' proprietary computer vision software (Fig. 6). Red light is used during the night cycle to detect the mice while maintaining a low subjective light level.

In Phenocube^®^ mice are tracked with both a micro transponder and through computer vision, which allows identification of individual mice in group settings. The standard test protocol comprises a conditional discrimination task, although other cognitive tasks can be program using the Intellicage software. Behavioral measures are obtained, every second for 24 hours and for several days, from the Intellicage unit and from the computer vision software and include measures of exploration, perseverative behavior, cognition, locomotion, rearing, climbing, social contact and interaction, and other measures.

Models of Huntington's disease are particularly relevant for demonstration of the utility of this system as they are characterized by altered cognition, motor activity, and circadian rhythms, all domains that can all be assessed simultaneously by the PhenoCube^®^ system. As an example, the R6/2 model shows blunted circadian amplitude in measures of activity as measured by locomotion, increased perseverative behavior as measured by repeat entries to a corner, and reduced rearing and climbing as age and pathology advance (Balci et al., 2013). PhenoCube^®^ can be used to track behaviors over 24 hours for multiple days at a time and thus can effectively pick up circadian deficits (Oakeshott et al., 2011), cognitive and social phenotypes that may be present only during the dark phase of the light cycle.

PhenoCube^®^ also adds the ability to score social behavior in an automated way. The system solves visual occlusions by tracking mice using both computer vision and telemetric data. We used two wild type strains, the C57 and BTBR strains from Jackson's labs as an example, as they have very different behavioral patterns. Phenocube's ability to capture social and activity behavior at the same time with high temporal precision allows detangling activity from true social behavior. Our results suggest, for example, that BTBR mice are not particularly less social than C57 mice, rather the latter are more active and aggressive (Fig. 6; Kabitzke, Mazzella & Brunner, unpublished). Such analyses of social behavior may prove of great importance for the understanding of social deficits in animal models of autism and schizophrenia.

## 5. Future Directions

In addition to phenotyping and drug development projects, PsychoGenics is working on the extension of the systems in two different directions. The first one involves integration of proteomics and genomics data with the observed behavioral signatures. We are developing methods to comb though the integrated dataset to find which are the best descriptors for a particular pharmacological or genetic signature. For example, using a neurodegenerative model, one can study in a comprehesive way both behavior and gene expression in the same subjects and then find a gene expression change that best correlates with cognitive deficits. Such gene may belong to a pathway that has not been investigated before. We hope that by finding a best omic predictor of functional deficits, novel unsuspected targets can be found.

PsychoGenics also developed a way to predict pharmacological effects of novel, never been tested, compounds through the use of a comprehensive model that identifies relationships between chemical features (custom high-resolution chemical fingerprints) and high-content behavioral readouts: Relationship Preserving Sample Generator (RPSG). RPSG is able to not only quantify non-linear relationships among all elements of high-content biological readouts and digitized representations of chemical structures (custom fingerprints), but also to subsequently generate tractable de-novo virtual chemical structures with the identified chemistry-biology relationships. Digitized chemical representation of those virtual compounds with the corresponding highest scored desired biological response are then used in the following ways:

- to compare to existing public and private libraries where compounds have been “digitized” the same way, allowing PsychoGenics to select the most promising molecules for testing (similar to Tanimoto similarity scoring except that we assign greater weights to the more discriminating fingerprint features);
- convert generated fingerprints back to the actual (novel) chemical structures using PsychoGenics' proprietary In-Silico Synthesis engine based on reaction database mining. We found that 94% of the resulting novel structures produced by RPSG are tractable (synthesizable).

**Figure 7:**
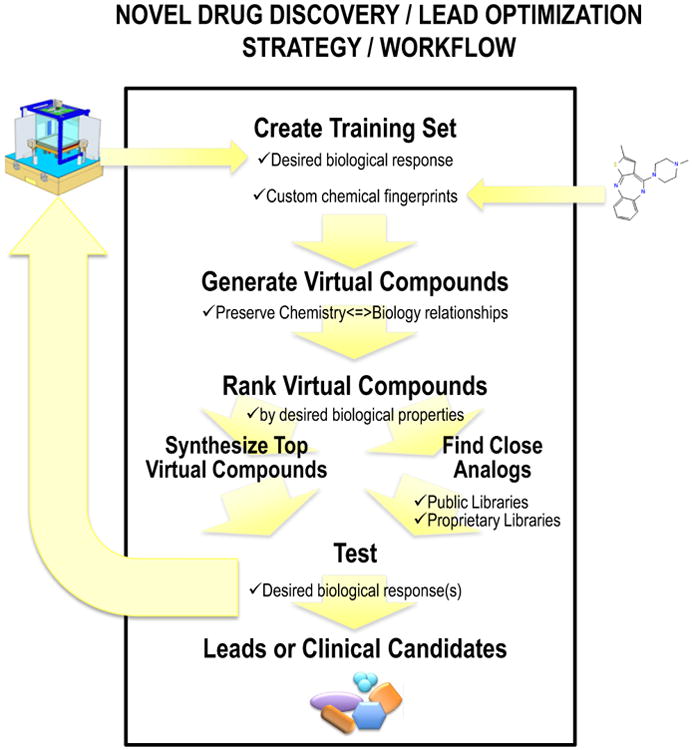
RPSG workflow in “Hit Generation” and “Lead Optimization” mode. The training set allows the RPSG algorithm to generate “Virtual compounds” which are ranked according to a desired biological response. Those compounds with the corresponding highest scored biological response are selected for testing and subsequent addition to the training set. The procedure is repeated until the most potent compound (which satisfies other selection criteria) is declared a “lead” or, later in the discovery process, an “optimized lead”.

Recent studies suggest that RPSG-produced hits have very high probability of showing in vivo activity (>90%). Thus, RPSG can be used to generate virtual libraries for screening (“hit generation” mode) when the supplied training set of molecules is chemically diverse (Fig. 7).

In addition, if the training set of molecules is an analog series of closely related chemical structures, RPSG can be used to optimize the biological ctivity and/orpotency, which makes it an ideal tool for lead optimization applications.

## Supplementary Material

1
