# Supplementary material for: Highthroughtput analysis of behavior for drug discovery
Source: Eur J Pharmacol. Author manuscript; Available in PMC 2016 Mar 5. (PMC4552324; doi:10.1016/j.ejphar.2014.11.047)
Supplement: 1 [file NIHMS661975-supplement-1.docx]

Supplementary Material - Methods

**Assurances**: This study was carried out in strict accordance with the recommendations in the Guide for the Care and Use of Laboratory Animals, NRC (1996). All protocols were approved by the Institutional Animal Care and Use Committee of PsychoGenics, Inc. (PHS OLAW animal welfare assurance number A4471-01), an AAALAC International accredited institution (Unit #001213).

## Animals

*CCI study*: Male C57/BL6 (8 weeks of age) mice from Taconic Laboratories (Germantown, NY) were used in the study. Upon receipt, animals were group-housed with 4 mice/cage. All animals were acclimated to the colony room for a week prior to surgery. All animals were examined, handled, and weighed prior to initiation of the study to assure adequate health and suitability. During the course of the study, 12/12 light/dark cycles were maintained. The room temperature was maintained between 20 and 23°C with a relative humidity maintained around 50%. Chow and water were provided ad libitum for the duration of the study. Following surgery, animals were single-housed. Animals were randomly chosen for treatment groups with the exception of the sham surgery animals.

*R6/2 studies*: R6/2 (R6/2 CAG 120, CHDI-81001000) transgenic heterozygous and wild type (WT) littermate control mice were obtained from Jackson Laboratories (Bar Harbor, ME; strain B6CBA-Tg[HDexon1]62Gpb/3J, stock number 006494). Mice were generated by crossing ovarian-transplanted females (from R6/2 CBAB6J female donors) with CBAB6F1J WT males. Genotype was determined by polymerase chain reaction (PCR) of tail-tip DNA at 15 days of age (Morton et al., 2000). CAG repeat lengths were measured by Laragen (Los Angeles, CA, USA) using standard protocols and Genemapper software as previously described (Menalled et al., 2009). *BTBR study*: BTBR T1tf/J and C57BL6/J mice were obtained from Jackson Laboratories (Bar Harbor, Me). Mice were pair-housed in OptiMouse cages and maintained on a 12:12 light:dark cycle. 8 BTBR mice and 8 age matched C57 control mice were tested in the PhenoCube apparatus at 6 months of age. Mice for PhenoCube^®^ were housed in genetically homogenous in OptiRAT^®^ cages (Animal Care Systems, CO).

**Methods**

**Sciatic Nerve Ligation Surgery**. Chronic constrictive nerve injury of the sciatic nerve was modified for mice according to Bennett and Xie (Bennett and Xie, 1988). Specifically, mice are anesthetized with isoflurane (2% in air). The left hind flank was shaved and sterilized and the mouse positioned on its side. The pelvic bone ridge was palpated and a vertical incision was made perpendicular to the long axis of the spine. Muscle was cut to expose the sciatic nerve. Retractors were used to open incision, centering the portion of the sciatic nerve to be ligated. The exposed nerve was carefully teased apart from the second layer of muscle, removing fascia lining.  Once the nerve was freed, hooked forceps were carefully passed underneath the nerve in order to pass 5 cm lengths of 6.0 chromic gut suture under the nerve (sutures are pre-soaked in saline to ensure softness). Sutures were positioned superior to the point where the nerve branches.  Each length of suture was used to make a loose ligation around the nerve (only tight enough to elicit a twitch).  All sutures were within a ½ cm range of each other.  The incision was closed in layers using 6.0 absorbable sutures for the muscle 5-0 non-absorbable suture for the skin.  Topical antibiotic ointment was applied to the sutured incision.  All subjects received buprenorphine (0.05 mg/kg in a volume of 10 ml/kg, i.p.) immediately before and 24 hrs after surgery. Each subject was monitored until it was awake and moving freely around the recovery chamber. Animals were then single-housed for the duration of the study and allowed to recover at least 1 week prior to testing.

**Von Frey Assessment.** Prior to dosing, mice were tested for their paw withdrawal threshold (PWT) using the von Frey filaments. Baseline and post-treatment withdrawal threshold values for non-noxious mechanical sensitivity will be evaluated using von Frey filaments (Semmes-Weinstein filaments, Stoelting) of varying stiffness. Each filament was presented perpendicular to the plantar surface with sufficient force to cause slight buckling against the paw, then held for approximately 6 seconds or until a positive response is noted. A positive response was defined as withdrawal from the von Frey filament. Confirmation of threshold was tested by examining the filament above and below the withdrawal response in a modified up-down method paradigm. If a response was positive, the next descending filament was tested. If the response was negative, the next ascending filament was tested. Each filament was applied 3 times. The responses from ipsilateral and contralateral paws were measured.

**NeuroCube**^®^ Mice were allowed to acclimate in the experimental room for 1 hour prior to test. Following acclimation mice were placed in the NeuroCube^®^ for and allowed to walk in the apparatus for 5 min. Subjects were returned to their colony room after testing.  Data collected included gait features (stride length, step length, base width, stride duration, stand duration and swing duration), speed, “paw features” (area of contact of the paw, perimeter of the paw print, minimal and maximal diameter of the paw image, intensity of the paw image), body motion (range of change of body dimensions, variability in body dimensions, range of body motion, variability in body motion), coordination (correlation of gait signals between pairs of paws) and paw positioning (paw position relative to body center, angles defined by every possible three-paw positions).

**SmartCube**^®^ Mice were taken in their home cage to the SmartCube^®^ suite of experimental rooms where they remained until they were placed in the apparatus. A standard SmartCube^®^ protocol for a single session lasts approximately 45 minutes. After the session, mice were placed back into to their home cage and returned to the colony room. Any abnormal behaviors were noted. Mouse behavior is captured by digital video using purposely designed hardware that presents multiple challenges in a single test session and is analyzed with computer algorithms.   Digital videos of the subjects are processed with computer vision algorithms to extract more than 1400 dependent measures including frequency and duration of behavioral states such as grooming, rearing, locomotor trajectories, posture, abrupt movements, stretched attend posture, and startle.

**PhenoCube**^®^**.** Experiments are conducted using modified Intellicage units (New Behavior, AG, Zurich, CH), each with a Day/Night Camera mounted on top of the cage for Computer Vision (CV) analysis. Spatial cues are added to the environment and as well as climbing structures. The cages are maintained at all times on constant red light. The light intensity under red light measures 7 lux and is sufficient to allow the camera to detect the mice while maintaining a low subjective light level for the mice. Prior to testing in PhenoCube^®^, mice are injected with sterile transponders (T-IS 8010 FDX-B, Datamars SA, Bedano, Switzerland) under 2% isoflurane inhalation anesthesia. The environment is enriched with a play tunnel, shredded paper, and a plastic bone. Food and water are available *ad libitum* with the exception of water deprivation 16 hours prior to PhenoCube testing. Temperature and humidity are controlled and monitored daily. *Habituation.* The Habituation phase is employed at the start of the experiment during which mice have access to all four of the corners. Intellicage corners are equipped with antennas to identify individual mice via their unique subcutaneous transponders, allowing for individualized behavioral testing parameters and data logging. As soon as any mouse enters a corner, both doors to the water open and remain open until the mouse leaves the corner. This phase of the experiment constitutes the “magazine training” and allows the mice to learn that water reinforcement is available in the corners. Basic activity data is collected during this phase of the experiments. The overall rate of corner visits and licking provide an insight into the general activity levels of the animals as well as their capacity to obtain water reinforcement, while evaluation of the overall rate of repeated visits (i.e. returning to the previously visited corner) gives some indication of the animals’ general tendency to persevere. *Alternation.* In the Alternation training protocol, the animals are required to visit two of the four corners in order to gain access to water. For each subject, two adjacent corners (active corners) along one of the shorter sides of the rectangular cage are contingently rewarded, while the other two (exploratory corners) are never rewarded. The Alternation protocol trains the animals to switch between the two active corners, only receiving reinforcement for alternating visits. Repeat visits to a corner are classified as incorrect, and mice do not receive a reward. Previous experiments at PsychoGenics have indicated that although mice do have some tendency to spontaneously alternate between foraging locations, their alternating behavior within the PhenoCube^®^ is significantly increased by the type of enforced Alternation training employed here, such that performance on this task does appear to be modulated by learning. Each corner contains two nosepoke recesses, one on each side, used to deliver water reinforcement during correct visits. Each active corner delivers water only on one side, with the rewarded side dependant on the specific identity of the current corner, such that if only the left side is rewarded in Active Corner 1 then only the right side is rewarded in Active Corner 2 and vice versa. No penalty is imposed for initially nosepoking on the incorrect side. *PhenoCube^®^ Computer Vision analysis.* Videos were processed using in-house developed segmentation and behavior detection algorithms. Single-subject tracking within a group of multiple mice was accomplished by computer vision-based subject shape analysis. All behavior endpoints were scored for individual mice. In addition to the main behaviors, i.e. locomotion, rearing, climbing and immobility, the principal social interaction behaviors were validated and defined as follows. Approach: Approach (Front, Rear): subject approaches a target and maintains it in view. Follow: subject trails a moving target and maintains it in view. Chase: subject trails a moving target at high velocity while maintaining it in its field of view. Interaction (Front, Rear): subject explores a target within 1.5 cm. The following behaviors were also assigned concurrently to the above measurements. Trajectory crossing: this behavior is scored when any part of a subject comes in contact with the target irrespective of the velocities and direction of the movement of the animals. Clustering: this behavior is scored when a subjects remains in Collision with a target for more than 0.75 seconds.
